# Ultrasound-Guided Lumbar Intradiscal Injection: A Case Report With a High-Resolution Ultrasound Image

**DOI:** 10.7759/cureus.49630

**Published:** 2023-11-29

**Authors:** Toru Omodani, Kieun Park, Hiroki Katayama

**Affiliations:** 1 Orthopaedics, Tokyo Advanced Orthopaedics, Tokyo, JPN; 2 Pain Medicine, PAKU Pain Clinic, Kobe, JPN; 3 Orthopaedic Surgery, Yokohama City University, Yokohama, JPN

**Keywords:** low back pain, ultrasound, ultrasound-guided injection, intradiscal injection, lumbar disc herniation

## Abstract

We present a case report demonstrating a new technique for intradiscal injection under ultrasound guidance in treating lumbar disc herniation. A 16-year-old female gymnast underwent this procedure and experienced relief from pain. Traditional methods have been noted for their technical challenges and potential risk of nerve root damage. In this case, our approach visualized the lateral side of the disc and improved needle visibility. This technique potentially offers a clearer high-resolution confirmation of the needle's position within the disc. It is considered that this technique is effective not only for performing precise injections but also for enhancing safety due to the clear depiction of the needle tip entering the intervertebral disc.

## Introduction

Lumbar disc herniation is a common disorder that occurs in the lumbar spine [1]. The typical symptom is lower back pain, and when the herniation protrudes posteriorly and compresses the nerve root, it can be accompanied by lower extremity pain [2]. Conservative treatments are generally considered to be effective. If rest, medication, and rehabilitation do not produce results, an intradiscal injection can be an option [3]. A study following a group of lumbar disc herniation cases over 13 years indicated that 14% of cases treated conservatively progressed to surgery, suggesting that intradiscal injections may be performed in at least an equivalent or greater proportion [4]. Intradiscal injections are typically performed under fluoroscopy, and reports of intradiscal injections under ultrasound guidance are extremely rare [5,6]. We report a case in which we performed a new method of intradiscal injection under ultrasound guidance for lumbar disc herniation.

## Case presentation

**Figure 1:**
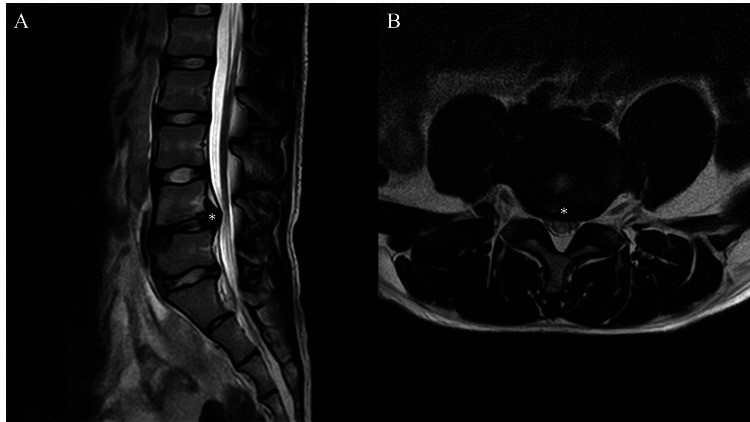
Magnetic resonance imaging Magnetic resonance imaging confirmed the herniation of the intervertebral disc between the fourth and fifth lumbar vertebrae (asterisk). A: sagittal plane, B: axial plane

A 16-year-old female gymnast began experiencing lower back pain during her competitions. One month after the onset of symptoms, she visited a local hospital and was diagnosed with lumbar disc herniation. She continued her training without taking a break but adjusted the training intensity and underwent rehabilitation. In rehabilitation, the focus was on strengthening the core muscles centered around the lumbar spine, as well as improving the range of motion in adjacent joints, such as the thoracic spine and hip joints, with the aim of reducing stress on the lumbar spine. However, the pain during her competitions did not improve, and her performance declined. Eight months after the onset of her symptoms, she visited our hospital. Her height was 156 cm, weight was 49 kg, and body mass index was 20.1. Magnetic resonance imaging confirmed degeneration of the L4/5 disc and its protrusion posteriorly (Figure 1). We decided to administer an intradiscal injection for her refractory lumbar disc herniation pain.

**Figure 2:**
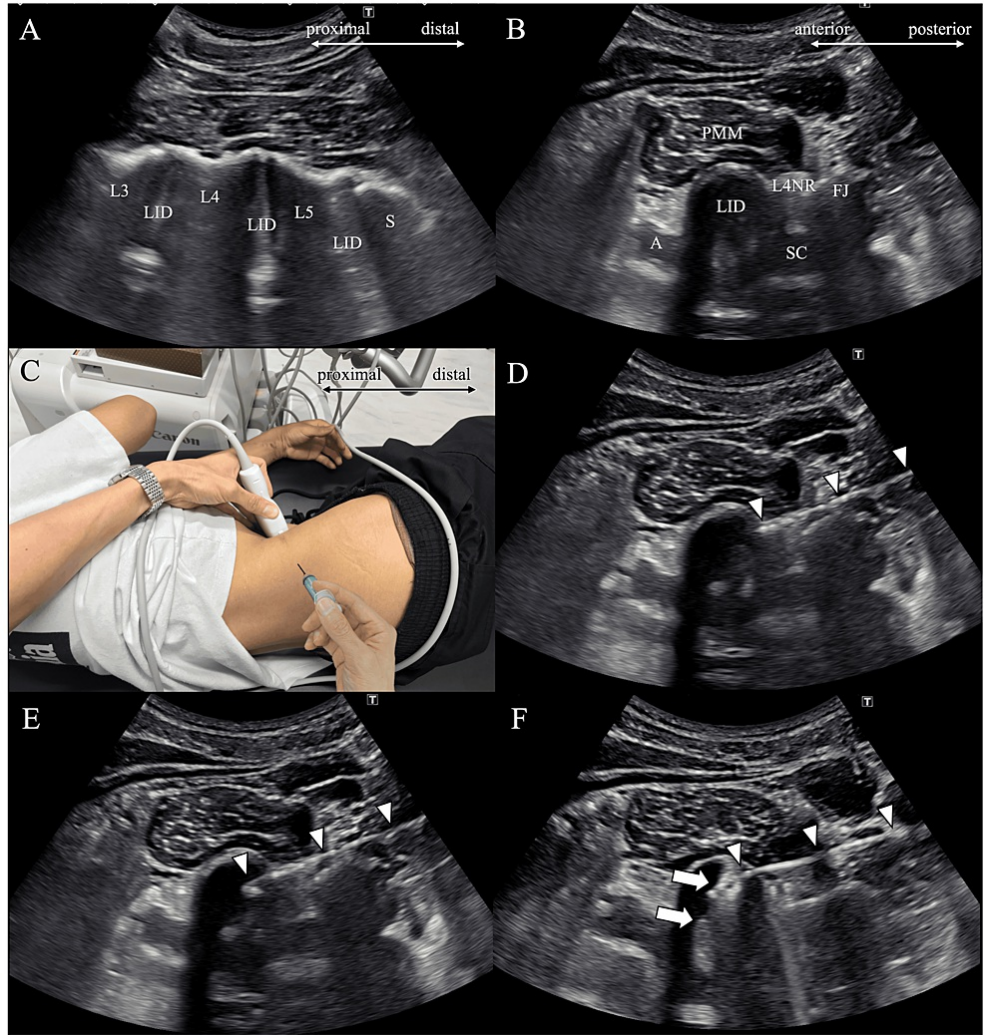
Ultrasound-guided lumbar intradiscal injection A: Identification of the lumbar vertebrae level in the long-axis view; B: Ultrasound image of the lumbar intervertebral disc and surrounding structures; C: The placement of the probe on the lumbar area and the entry point and direction of the needle; D: The needle reached the outer edge of the intervertebral disc; E: The needle tip was inserted into the intervertebral disc; F: Medication was injected into the intervertebral disc; LID: Lumbar intervertebral disc SC: spinal canal, A: aorta, PMM: psoas major muscle, FJ: facet joint, L4NR: fourth lumbar nerve root, arrowheads: needle, arrow: medication injected into the intervertebral disc

The patient was placed in a lateral decubitus position on the bed with her right side up. It was necessary for the patient to be in a lateral decubitus position to apply the probe from the anteromedial side of the body and administer the injection from the posterolateral side. To stabilize the patient's position, a pillow was placed under the head, and a cushion was positioned between the legs. The ultrasound probe was placed longitudinally on the anterolateral aspect of the right lumbar region. The probe was positioned at this angle to clearly depict the needle entering the intervertebral disc. Using the sacrum as a reference, the level of the lumbar vertebrae was identified (Figure 2A). With the L4/5 intervertebral disc visualized in the center of the screen, the probe was rotated 90 degrees to depict the axial view of the disc (Figure 2B). A 22-gauge, 89-mm needle was inserted from the posterolateral aspect of the lumbar region (Figures 2C, 2D). During the injection procedure, an area with a diameter of approximately 10 cm around the needle insertion point was disinfected with iodine. The specified needle was chosen to ensure sufficient length to reach the intervertebral disc because a needle with too fine a diameter might not allow for the injection of medication into the disc. During this, the needle entry direction and the probe orientation were aligned as perpendicular as possible, and the needle entry point into the skin was placed as far away from the probe as possible. The needle tip's position within the nucleus pulposus of the intervertebral disc was confirmed on the ultrasound image (Figure 2E). It was confirmed that the needle tip was positioned at the center of the intervertebral disc. For this confirmation, it was necessary to obtain high-quality images of both the needle tip and the intervertebral disc. A total of 2 ml of a mixed solution containing 1% lidocaine (1 ml) and dexamethasone (3.3 mg) was injected into the disc (Fig 2F). For the injection, a local anesthetic was chosen to avoid pain associated with increased intradiscal pressure, and a steroid was selected for its anti-inflammatory properties. The ultrasound machine used was the Aplio i700 manufactured by Canon Medical Systems, Tochigi, Japan, and an 8MHz convex probe was employed. In performing delicate procedures in the deep region of the lumbar area, this specific machine and probe combination was chosen to obtain high-resolution images with a wide field of view. There were no complications associated with the injection. The patient resumed training the next day. Upon resuming athletic activities after the injection, the patient was instructed to ensure there were no side effects such as worsening back pain or the onset of leg pain that emerged post-injection. After the injection, the lower back pain gradually improved. The numerical rating scale during competition decreased from 8 to 2 out of 10 one week after the injection, and her performance in competitions improved.

## Discussion

In this case, an intradiscal injection under ultrasound guidance was performed for lumbar disc herniation. This technique is a novel method that has not been reported previously.

Wu et al. were the first to report intradiscal injections under ultrasound guidance [5]. The path through which the needle enters the disc was an extremely narrow space between the lumbar nerve root as it exited the foramen and the facet joint. This presented technical challenges and posed a potential risk of nerve root injury. Lam et al. reported a technique that approaches the disc more laterally compared to the earlier study [6]. However, their report was limited to injections in the intervertebral disc between the fifth lumbar vertebra and the sacrum. Additionally, the orientation of the needle in the ultrasound image was steeply directed towards the deeper tissues. From the perspective of visualizing the needle, the technique seemed challenging, and there was no clear image on the ultrasound showing the tip of the needle entering the disc.

In this case, the probe was applied from the anterolateral aspect of the lumbar region to visualize the lateral part of the disc. To insert a needle into the intervertebral disc, approaching from the front, where abdominal organs are present, or from the posterior and posterolateral, where nerve roots and the spinal canal are located, poses the risk of organ or nerve damage. Therefore, ultrasound imaging was used to visualize the lateral side of the disc, a region with a comparatively lower risk of tissue damage, to insert the needle tip. While this method is considered to be effective, it is necessary to exercise caution to prevent the needle tip from mistakenly advancing toward the anterior aspect of the body and into the abdominal cavity. Moreover, by placing the needle entry point away from the probe and aligning the needle entry direction and the probe orientation as perpendicularly as possible, the visibility of the needle on the ultrasound image was enhanced. The most critical aspect of this technique is the position of the probe and the insertion angle of the needle to clearly depict the needle tip. These modifications were considered effective not only in facilitating precise injections but also in enhancing safety.

Although the patient in this case was thin, there is a possibility that image quality may diminish in larger patients. In such cases, greater attention should be given to the visualization of the intervertebral disc and the needle.

## Conclusions

For lumbar disc herniation, we performed a new method of intradiscal injection under ultrasound guidance, a technique not previously reported, and achieved favorable results. It is considered that this technique is effective not only for performing precise injections but also for enhancing safety due to the clear depiction of the needle tip entering the intervertebral disc. The novelty of this method lies in obtaining a high-resolution and clear image of the needle tip entering the intervertebral disc. The introduction of this technique, which allows for the high-quality visualization of both the intervertebral disc and the needle tip while maintaining safety, has the potential to boost clinical practice and research in the field by promoting the implementation of ultrasound-guided intradiscal injections. As this report is merely a case study, widespread adoption of this method will require cadaver studies to prove its accuracy and clinical research to demonstrate its efficacy.
